# Iron Pathways and Iron Chelation Approaches in Viral, Microbial, and Fungal Infections

**DOI:** 10.3390/ph13100275

**Published:** 2020-09-25

**Authors:** Ravneet Chhabra, Aishwarya Saha, Ashkon Chamani, Nicole Schneider, Riya Shah, Meera Nanjundan

**Affiliations:** Department of Cell Biology, Microbiology, and Molecular Biology, University of South Florida, Tampa, FL 33620, USA; chhabra@usf.edu (R.C.); alsaha@usf.edu (A.S.); ashkon@usf.edu (A.C.); npschneider@usf.edu (N.S.); riya@usf.edu (R.S.)

**Keywords:** hepatitis C virus, human immunodeficiency virus, gram-negative bacteria, gram-positive bacteria, fungi, iron chelators, iron uptake pathways

## Abstract

Iron is an essential element required to support the health of organisms. This element is critical for regulating the activities of cellular enzymes including those involved in cellular metabolism and DNA replication. Mechanisms that underlie the tight control of iron levels are crucial in mediating the interaction between microorganisms and their host and hence, the spread of infection. Microorganisms including viruses, bacteria, and fungi have differing iron acquisition/utilization mechanisms to support their ability to acquire/use iron (e.g., from free iron and heme). These pathways of iron uptake are associated with promoting their growth and virulence and consequently, their pathogenicity. Thus, controlling microorganismal survival by limiting iron availability may prove feasible through the use of agents targeting their iron uptake pathways and/or use of iron chelators as a means to hinder development of infections. This review will serve to assimilate findings regarding iron and the pathogenicity of specific microorganisms, and furthermore, find whether treating infections mediated by such organisms via iron chelation approaches may have potential clinical benefit.

## 1. Introduction

### 1.1. Iron, an Essential Element for Survival of Both Hosts and Microorganisms

Iron is a key element needed to support fundamental cellular processes including oxygen transport, DNA replication, transcription, and metabolic processes in many living organisms [1,2]. It is also essential to support the growth, virulence, and pathogenicity of microorganisms such as viruses, microbes, and fungi [3,4], which can acquire iron from within its host environment.

Dietary iron in the host can be obtained in the form of heme from various sources including red meat, seafood, and poultry [5]. This heme iron is absorbed into cells through a mechanism that involves the Heme Carrier Protein (HCP1), a proton-coupled folate transporter (PCFT) [6]. Non-heme iron can be obtained predominantly from plant sources [6]. Non-heme iron absorption into cells can occur either as transferrin bound iron (TBI) or non-transferrin bound iron (NTBI) [7,8,9]. The absorption of iron into the bloodstream is primarily regulated by hepcidin (HAMP), a liver secreted peptide hormone [10].

In an adult human, hemoglobin from red blood cells (RBCs) contains approximately two-thirds of the total iron present in the body (~3–4 g) [5]. Not only is iron stored in liver cells and macrophages bound to ferritin, but it is also found in myoglobin of muscle cells [5]. Further, phagocytosis of RBCs by macrophages leads to the release of iron from hemoglobin and serves as a crucial source of iron [11]. Heme iron degradation involves the action of heme oxygenase 1 (HO-1) or heme oxygenase-2 (HO-2) [12], whereby the released free iron becomes part of the labile iron pool (LIP), a redox-active pool of intracellular iron [13].

The specific mechanisms that support the ability of microorganisms such as viruses, microbes, and fungi to uptake iron from sources in the host are discussed in this review, along with the effect of iron chelators which may potentially antagonize their growth and virulence.

### 1.2. Host Cell Iron Metabolic Pathway

It is well established that deregulated iron control can lead to detrimental effects on survival [1,2]. Since redox-active iron is a catalyst in electron transfer and free radical reactions, excessive amounts of free iron can deteriorate cell health (e.g., DNA damage, lipid peroxidation, and protein oxidation) [14]; therefore, a tightly regulated system is essential to appropriately balance intracellular iron levels [15]. This mechanism of control has been deciphered and involves a large array of mediators described below.

Transferrin, a carrier glycoprotein which binds to iron (as Fe^3+^-bound complex (TBI)), facilitates the transport of iron into cells via the transferrin receptor (CD71) [16]. Cellular entry of TBI occurs via an endocytic process which is followed by the release of iron from transferrin due to the reduced pH of the endosomal compartment. Subsequently, STEAP3 (Six Transmembrane Epithelial Antigen of Prostate 3) mediates reduction of the Fe^3+^ (ferric) to the Fe^2+^ (ferrous) form [17]. Once reduced, the iron is released from the endosomal compartment to the cytosol via endosomal DMT1 (Divalent Metal Transporter 1) [18].

The divalent metal transporter ZIP8 (Solute carrier family 39 member 8 (SLC39A8)) is one way through which NTBI can enter cells [19]. Another mechanism underlying NTBI uptake, specifically into small intestinal cells, involves the reduction of ferric iron via duodenal cytochrome b (DCYTB) [20] followed by its transport via cell surface localized DMT1 [21,22].

The imported iron (from either NTBI or TBI) can either (1) be stored in a complex with ferritin (FTN), (2) be added to the labile iron pool (LIP), (3) be exported extracellularly via ferroportin (FPN), or (4) be integrated within key enzymes involved in regulating cellular metabolic processes [23]. Extracellular export involving FPN is the only means of exporting iron out of cells and its levels are regulated by HAMP [1,2,23,24].

Iron-binding proteins in the host plasma (e.g., transferrin, haptoglobin, hemopexin, lactoferrin, lipocalin-1, and lipocalin-2) have the ability to sequester iron from various sources; their iron binding capacity can contribute to reducing the availability of extracellular iron that may support the growth and virulence of microorganisms within this environment [25]. Withholding of intracellular host iron (e.g., reduced iron uptake, increased storage of iron in ferritin, increased export of iron via FPN) may also contribute to reducing intracellular microorganismal growth [25]. Hence, tight regulation of the above described iron metabolic pathways is critical in mediating the balance between microorganisms and the host.

### 1.3. Iron Chelation and Associated Risks

In multiple diseases, iron chelation has been explored as a therapeutic regimen to reduce iron levels to promote health [26]. Efficacy of iron chelators depends on high membrane permeability and effectiveness of oral administration [27]. There are two major classes of iron chelators: (1) naturally occurring, e.g., Epigallocatechin-3-gallate (EGCG, found in green tea), phytic acid, curcuminoids, and (2) synthetic, e.g., Deferiprone (DFP or L1), Deferasirox (DFRA or DFX), 8-hydroxyquinoline derivatives such as VK-28 and M30 [28]. Some chelators are derived from bacterial sources including Desferioxamine (also known as deferoxamine (DFO), produced by *Streptomyces pilosus*) and Desferrithiocin ((DFT), a tridentate siderophore, produced by *Streptomyces antibioticus*) [28,29,30]. Moreover, phytochelators, obtained from plant components including vegetables and fruits, elicit iron-chelating activities; polyphenols are one such class with the ability to chelate iron with high affinity and promote health [31,32]. Mammalian-derived physiological iron chelators include (1) transferrin, found in blood plasma and involved in body-wide iron transport and (2) lactoferrin, enriched in neutrophils and in bodily secretions [32,33,34]. Not only do these physiological iron chelators bind iron but they also elicit anti-microbial activities to hinder the propagation of microorganisms [32,33,34].

A subset of FDA-approved iron chelators includes DFO, DFRA, and DFP [26] and these have different means of patient administration (e.g., oral versus intravenous) with divergent efficacies and blood brain barrier accessibilities [32]. Some of the above described iron chelators have shown success in combinatorial treatment strategies with antibiotics, anti-virals, and anti-fungals. However, the clinical applications of these iron chelators are noted to be associated with health risks. DFO, the first clinically applied iron chelator which is administered intravenously, is associated with side effects affecting vision, hearing, and kidney function; further, in some cases, *Yersinia* and *Klebsiella* infections may develop [35]. DFP, administered orally, is also associated with some health risks including alterations within the immune system (e.g., neutropenia, agranulocytosis, thrombocytopenia) in addition to arthropathy and adverse effects on liver function [35]. DFRA, also administered orally, is associated with adverse effects on the liver, digestive system, and skin [35]. Furthermore, the application of these iron chelators may result in anemia [35].

**Figure 1:**
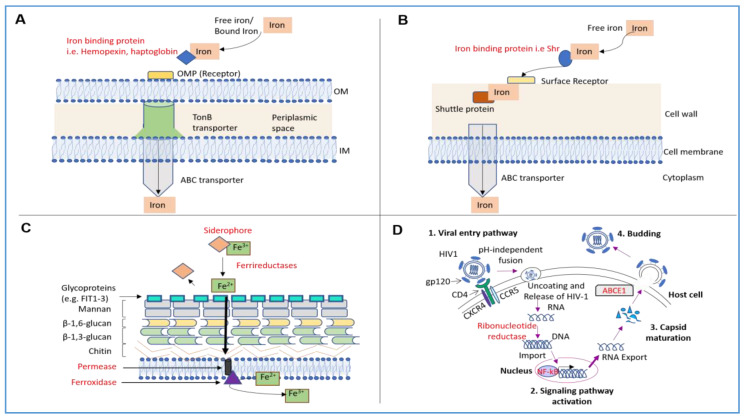
Mechanisms of Iron Acquisition in Bacteria and Fungi as well as Mechanisms of Iron Utilization in Viruses. (**A**) In Gram-negative bacteria, the iron acquisition involves uptake of free iron from the extracellular environment. Specific proteins that bind heme or hemoglobin (e.g., hemopexin or haptoglobin) are secreted from the bacteria, to then bind to free iron or heme [36]. These complexes then interact with outer membrane protein (OMP) receptors on the surface of the outer membrane (OM) of the bacterial cell wall [37]. The iron is then moved to the TonB–dependent receptor complex [36]; after the iron reaches the ABC transporter, it finally passes the inner membrane into the bacterial cytoplasm [37]. (**B**) In Gram-positive bacteria, secreted specific iron/heme binding proteins interact with heme [37], following which, the iron binding protein localizes to the surface of the bacterial cell wall to bind to a specialized cell surface receptor [38]. Next, specific permeases enable the translocation of this complex across the bacterial cell wall [39]. The iron-bound molecule is subsequently transferred to a shuttle protein that then guides the iron into the ABC transporter, an integral membrane protein [39]. The ABC transporter translocates the iron-bound molecule into the bacterial cytoplasm [39]. (**C**) In fungi, the iron uptake system involves reductive and/or non-reductive mechanisms [40]. In the reductive iron assimilation pathway, iron acquisition is initiated via siderophores which bind to ferric iron; the iron is subsequently reduced via ferrireductases and released from the siderophores as the ferrous form [40]. The iron is then translocated to glycoproteins on the surface of the fungal cell wall and the uptake of iron is mediated by permeases followed by oxidation of ferrous to ferric iron via ferroxidase [41]. (**D**) HIV replication is shown herein, as an example. There exists multiple pathways for viral-host cell entry; specifically, HIV entry into a host cell requires interaction of the HIV gp120 with the CD4 receptor (associated with CCR5 or CXCR4 co-receptors) [42]; this is followed by the pH-independent fusion of the virus with the host cell membrane. Subsequently, the viral RNA is released into the cell and reverse transcribed into DNA via ribonucleotide reductase (RNR) and then imported into the nucleus [43]. Iron can increase NF-kB activation leading to upregulation of HIV gene expression. Finally, the iron-dependent ATPase transporter ABCE1 promotes HIV-1 capsid maturation [4].

In this review, we summarize findings involving the application of iron chelators primarily in the context of infectious diseases (see Figure 1 for an overview of iron acquisition pathways).

## 2. RNA-Based Viral Infections

Overview—Viruses have adapted themselves to use host cell components to propagate. In contrast to Hepatitis C Virus (HCV) and Human Immunodeficiency Virus (HIV) which utilize SR-B1 and CD4 receptor, respectively, for host cell entry, New World Arenaviruses enter host cells by binding to transferrin receptors, a host cell receptor also responsible for iron uptake [42,44]. To support viral replication, ribonucleotide reductase (RNR), responsible for generating deoxyribonucleotides from ribonucleotides, is essential and requires iron to support its activity [45]. Host cell signaling pathways may also respond to altered iron availability (e.g., increased NF-kB activation) to support viral infections [46]. Likewise, the maturation of viral particles requires the iron-binding ATP binding cassette subfamily E member 1 (ATPase ABCE1) [4,47]. Therefore, iron appears to be a critical element to support multiple elements supporting propagation of a virus including its entry, replication, and maturation. Evidence supporting the use of iron chelators to antagonize viral propagation is presented herein.

### 2.1. Hepatitis C Virus (HCV)

Viral Genome and Structure—Hepatitis C Virus is a member of the *Flaviviridae* family and is a single-stranded positive sense virus with a diameter of 50–80 nm [48]. Its genome is comprised of 10 elements and codes for structural and non-structural components: (1) Core, capsid, (2) E1 and E2, envelope glycoproteins, (3) p7, viroporin (this protein forms pores in the host cell membrane to enable viral propagation) [49] and assembly factor, (4) NS2, autoprotease and assembly factor, (5) NS3, serine protease and helicase, assembly factor, (6) NS4A, NS3 protease co-factor, (7) NS4B, scaffold protein of the replication complex, (8) NS5A, regulator of replication and viral assembly, and (9) NS5B, DNA-dependent RNA polymerase [50]. Its entry into host cells is a complex process and involves a multitude of host cell factors such as CLDN1 (Claudin 1), OCLN (Occludin), CD81 (Cluster of Differentiation 81), and SRB1 (Scavenger receptor class B type 1) [48].

Health Implications and Current Treatments—HCV infections are associated with inflammation potentially leading to liver fibrosis, cirrhosis, and hepatocellular carcinoma [51]. Although HCV is considered curable, the health implications and risk of hepatocellular carcinoma remain a concern [51]. Interferon (IFN) has been the sole treatment regimen for HCV infected patients with only 15%–20% eliciting a sustained virological response following 11 months of treatment [52]. However, ribavirin, an immunomodulating agent described as a synthetic guanosine analogue, not only directly inhibits viral replication but also promotes efficacy of IFN, relapse response in patients infected with HCV, and a sustained virological response [53,54]. Specifically, recent data demonstrate that ribavirin promotes the JAK-STAT signaling cascade to enhance anti-viral responses against HCV [53]. Additional pathways that are activated in response to HCV include EGFR and TGF-β activation, which may contribute to disease progression and potentially offer additional targets for therapy [55]. Identification of further therapeutic regimens to combat the health complications of HCV are needed.

Iron Contribution to HCV Infections—In liver-biopsy specimens from infected HCV patients, a positive correlation between hepatic iron content and HCV was reported [56]. Efforts were also made to reduce iron levels by phlebotomy in infected HCV patients to improve outcomes. For example, in male patients in whom anti-viral therapy was ineffective, phlebotomy (administered every 1 to 3 months over a 2 year period) improved the liver histology [57]. In HCV patients characterized by elevated levels of serum alanine aminotransferase (ALT, a marker of liver damage [58]) and iron deposits in their livers, phlebotomy (performed every week or monthly over a 9 month period) improved their liver function [59]. This is supported by an independent study in which patients (resistant to IFN-α with no abnormal profile of liver iron content) responded positively to phlebotomy (over a 2 week period) with improved ALT activity [52]. However, it remains unclear whether phlebotomy can reduce HCV viral load since it was either not reported or not determined in the above described studies [52,57,59].

In in vitro studies, supplementation with FeSO_4_ for two days was found to increase the replicative capacity of HIV (as measured by quantification of the viral RNA) in hepatocytes [60]. Further, the iron-induced translation of HCV was mediated by factors involved in the initiation of translation including eIF3 (translation initiation factor 3) in HepG2 cells [56] and La proteins (which bind to the internal ribosome entry site (IRES) to regulate initiation of translation of HCV RNA) [61,62,63]. With respect to iron chelators, DFRA was reported to antagonize HCV-induced upregulation of these translation initiation factors in Huh-7 cells [63]; further, antisense phosphorothioate oligodeoxynucleotides targeting these initiation factors reduced iron-induced HCV translation in Huh-7 cells [63]. In contrast, iron, presented as a complex with salicylaldehyde isonicotinoyl hydrazine (lipophilic tridentate iron chelator, Fe-SIH), could mediate anti-viral effects by reducing expression of viral proteins (NS3 and Core) in Huh7.5.1 cells [64].

Although it has been suggested that therapies that reduce iron levels could be utilized as adjuvant to existing HCV therapies [60], additional studies are needed to provide support for the use of iron chelators as an adjuvant therapeutic regimen in HCV-infected patients.

### 2.2. Human Immunodeficiency Virus (HIV)

Viral Genome and Structure—Human immunodeficiency virus is a member of the *Retroviridae* family and is a single stranded RNA virus with a diameter of 100 nm [43]. HIV contains 9 elements in its genome: (1) *Gag*, codes for the core structural proteins (p24, p7, and p6) and the matrix (p17), (2) *Pol*, codes for viral replication enzymes including reverse transcriptase, integrase, and protease, (3) *Vif*, encodes a protein that promotes infectivity of viral progeny, (4) *Vpr*, codes for a protein that causes cell cycle arrest, (5) *Vpu*, codes for a protein involved in the release of the viral particle, (6) *Tat*, codes for a protein which is involved in HIV gene expression, (7) *Rev*, codes for a protein that allows the export of the RNA from the nucleus into the cytoplasm, (8) *Env*, encodes the glycoproteins in the envelope (gp120 and gp41), and (9) *Nef*, codes for a protein that can modulate signaling and promote viral budding [43]. The viral budding process leads to host cell lysis; however, during latency, the viral DNA lies dormant in the nucleus of specific host cells including CD4+ T Cells, which is referred to as a cellular reservoir [65,66].

Health Implications and Current Treatments—HIV can lead to acquired immunodeficiency syndrome (AIDS), a terminal stage of HIV-infections, in which patients are afflicted by opportunistic infections and cancer [67]. There is presently no cure for these patients, who are characterized by a progressive reduction of white blood cells (CD4+ T cells) as a result of the deterioration of tissues that generate lymphocytes (e.g., bone marrow and thymus) [68] and opportunistic infections (e.g., Candidiasis, Cryptococcosis, amongst others) [69] which eventually lead to mortality, if left untreated [70]. Current treatment regimens include anti-retroviral therapy (ART) which integrates three drugs including two nucleoside reverse transcriptase inhibitors, which compete with deoxynucleotides incorporated into DNA that is being replicated (e.g., emtricitabine and tenofovir), and one non-nucleoside reverse transcriptase inhibitor, integrase inhibitor, or protease inhibitor (e.g., raltegravir) [71]. Regrettably, the persistence of the virus in a cellular reservoir in latent form hinders its complete elimination even with ART treatment regimens [70]. Thus, identification of other targets to effectively deplete these reservoir holding cells are direly needed for an effective cure.

Iron Contribution to HIV Infections—In patients with HIV infections, excessive iron content in their serum [72], tissues (e.g., bone marrow [73,74], brain white matter [75,76], muscle [77]), and their cells (macrophages and microglia) [75] is suggested to contribute to the pathogenesis of the disease [78]. Further, a link between iron and HIV disease progression was identified in a male HIV patient affected by the iron overload condition, hereditary haemochromatosis (characterized by a mutation at C282Y in the *HFE* gene); specifically, a major reduction in viral particles was noted following an extensive 18-month phlebotomy period [79].

At a cellular level as determined from in vitro studies, the addition of exogenous iron or modulation of key modulators of host iron metabolic pathways could alter HIV-1 replication and transcription. Specifically, overexpression of FPN1, the iron export receptor, decreased transcription of HIV-1 in HEK293 cells [80]. In contrast, the addition of HAMP, which downregulates FPN1 and increases LIP, antagonized viral transcription in promonocytic cells as well as macrophages and CD4+ T cells [80,81]. Under conditions of excess ferrous sulfate heptahydrate, the survival of HIV-infected T-lymphoid CEM-syncytial sensitive cells was reduced and correlated with elevated viral replication (p24, which is a core protein of HIV encoded by *gag*) and reverse transcriptase activity in the cell supernatants [82]; these cellular responses were opposed by the iron chelator, DFO [82].

The mechanisms underlying these iron-associated cellular effects on HIV-1 transcription were elucidated to be mediated through NF-kB (a transcription factor that plays a role in regulating multiple cellular activities), which binds to the long-terminal repeat sequence of HIV (the control center for regulation of HIV gene expression with binding sequences for both host and viral proteins) on NF-kB response elements [83]. This pathway could be opposed by the iron chelator, DFO, in specific cell types (namely, U1 (an HIV-infected U937, a pro-monocytic myeloid leukemia cell line) and ACH-2 cells (HIV-infected acute lymphoblastic leukemia T cell line)); this was demonstrated via a gel shift assay in which DFO led to a marked reduction in NF-kB retardation complex [46].

Inhibition of the iron-dependent DNA replication enzyme, RNR, with iron chelators (DFRA and DFP) in lymphocytes could also alter HIV viral replication [84,85]. Bleomycin (BLM, an antibiotic isolated from *Streptomyces verticillus* which forms iron complexes generating ROS that leads to base modifications in the viral DNA) could also reduce the replicative capacity of HIV without affecting cellular viability in PBL (peripheral blood lymphocytes); whether the effects of BLM is due to iron-chelation activity is unclear [85]. Furthermore, BLM and the commonly used iron chelators DFO and DFP were capable of reducing the expression of the viral capsid core protein (p24) in macrophages and PBL [85]. Iron chelators with comparatively higher affinity for iron, CP502 and CP511 (bidentate chelators of the 3-hydroxypyridin-4-one family) were also effective in reducing viral replication (viral capsid core protein p24) by altering cellular viability (^3^H-thymidine) of peripheral blood lymphocytes [86].

In vitro cell studies were performed to identify changes in the expression and activities of cell cycle mediators by iron chelators in HIV-1 infected cells. Specifically, the iron chelators DFO and 311 (2-hydroxy-1-naphthylaldehyde isonicotinoyl hydrazone) reduced HIV-1 viral transcription by modulating protein expression of cyclin-dependent kinases (e.g., CDK2, a mediator in cell cycle progression) in human lymphoid CEM cells [87]. Moreover, 311 and yet another iron chelator, ICL670 (4-[3,5-bis-(hydroxyphenyl)-1,2,4-triazol-1-yl]-benzoic acid), hindered HIV-1 transcription of the *Tat* gene by reducing CDK2 and CDK9 kinase activity on specific target proteins including cyclin T1 and the C-terminal domain of RNA polymerase II in multiple cell types including HeLa-CD4-LTR-β-gal cells, 293T cells, and CEM cells [87]. This finding is of particular importance since the Tat protein plays a key role in activating the latent virus by physically binding to CDK2 and CDK9 complexes [87]. Other novel iron chelators, including Phenyl-1-Pyridin-2yl-Ethanone-Based, inhibited CDK2 activity, reduced CDK9 levels, and increased IkBα and cytoplasmic NF-kB to mediate reduction in HIV-1 transcription of the B subtype in infected T cells [88].

Although there appears to be an abundance of data supporting the use of iron chelators in HIV-1 infected cell lines, additional research is needed to enable the use of iron chelators in clinical treatment strategies.

## 3. Bacterial Infections

Physical Characteristics of Gram-negative and Gram-positive Bacteria with Relevance to Iron Uptake—It is well established that iron acquisition is critical in promoting the growth and virulence of numerous pathogenic bacteria [32]. Since antibiotic resistance is of major concern in the treatment of pathogenic bacterial-induced infections, novel treatment agents are direly needed. Both Gram-negative and Gram-positive bacteria have well adapted strategies for iron uptake including specific surface chaperone proteins for acquiring heme, receptors, and ABC (ATP-binding cassette) transporters for membrane translocation, as described in detail in [89,90,91]. The iron uptake mechanisms differ between Gram-negative and Gram-positive bacteria and these differences are contributed by their physical characteristics of the outer cell membrane [92]. Specifically, Gram-positive microbes are characterized by a thick layer of peptidoglycan incorporated within the cell wall along with the extracellular exposure of teichoic acids and lipoteichoic acids as well as a diminished periplasmic space volume [90]. In contrast, Gram-negative microbes contain both an outer and inner membrane along with a larger periplasmic space volume [92]. Iron uptake mechanisms that are common across both Gram-negative and Gram-positive microbes include the involvement of ABC transporters [93].

Iron Uptake Pathway in Gram-negative Bacteria—Greater than 30 outer membrane protein (OMP) heme receptors (involved in transporting heme intracellularly) have been characterized across a wide variety of Gram-negative microbes [94]. Some specific molecules that are engaged in this process include those involved in the direct binding of heme (e.g., hemopexin) or hemoglobin (e.g., haptoglobin) to OMP receptors on the bacterial cell wall [36,37]. After delivery of the heme to the periplasmic membrane, the heme is then transferred via the ABC transporters to the cytoplasmic compartment [37]. Another acquisition mechanism involves hemophores, which interacts with free heme in the external environment and transports it to the surface of the bacterial cell membrane [37]. When the heme is transported to the bacterial cell surface, it then interacts with the TonB dependent transport pathway [37,95,96]. As a specific example, the heme acquisition system A (HasA) participates in the heme-uptake process in the pathogenic microbe, *Pseudomonas aeruginosa*, which we discuss in greater detail below [97]. Another example includes the heme/hemopexin utilization (HxuA) pathway which is involved in pathogenic microbes that are deficient in heme production [98]. The ferric uptake regulator (Fur) protein, a transcriptional regulator of iron uptake genes, is a key mediator of iron regulation in Gram-negative microbes as well [99].

Iron Uptake Pathway in Gram-positive Bacteria—In contrast to Gram-negative microbes, far fewer details have been uncovered with respect to the mechanisms underlying heme uptake in Gram-positive microbes. However, the HemT-like lipoprotein, HmuT, has been identified to participate in this process, specifically in *Corynebacterium diphtheriae*, a well-studied Gram-negative microbe [100]. The components of the ABC transporter pathway in *Streptococcus* *pyogenes*, another well-studied Gram-negative microbe, involves the Shr protein (which binds heme), the streptococcal heme-binding protein Shp (which relays the heme for transport across the bacterial envelope), SiaA (which is the heme-binding lipoprotein), SiaB (membrane permease), and SiaC (ATPase) [38,39]. A more well understood mechanism of the pathogenic bacterium, *Staphylococcus Aureus*, has been identified which uses hemolysins in its pursuit to acquire bound heme [101].

### 3.1. Pseudomonas Aeruginosa: A Gram-Negative Microbe Associated with Wound Infections and Cystic Fibrosis

Bacterial Features—*Pseudomonas aeruginosa*, a multi-drug resistant pathogen, has a genome size of 5.5–7 Mbp with the capacity to express genes underlying resistant phenotypes [102]. Together with complex metabolic processes, these features support its propagation in unfavorable environments [103]. The pathogenicity of *P. aeruginosa* is mediated by structural components including flagellum and pili as well as cell surface glycolipids and lectins that are involved in bacterial movement and adhesion to host cells [104]. Further, secretion of virulence factors is mediated by quorum sensing pathways leading to secretion of elastases (proteases) into the host environment [104]. In addition, *P. aeruginosa* is capable of injecting cytotoxins into host cells [104].

Contribution of Iron—Several iron uptake mechanisms are involved in mediating *P. aeruginosa* growth properties [105]. The pyoverdine (Pvd) and pyochelin (Pch) siderophores (of which pyoverdine has a higher iron affinity) are involved in the movement of extracellular iron into *P. aeruginosa* [106,107] using FptA and FpvA (outer membrane proteins) [108]. The addition of Pch with *P. aeruginosa* injected intraperitoneally in Swiss–Webster mice resulted in increased virulence [109]. Using immunosuppressed mice, mutant strains of *P. aeruginosa* deficient in Pvd or Pch/Pvd inoculated intranasally, elicited reduced growth in the pulmonary tissue coinciding with decreased virulence [110].

In wounds infected with *P. aeruginosa*, the rate of repair is diminished in multiple animal models (e.g., rabbit, murine, pig in vivo models) [104]. Specifically, in a murine wound model (in which a muscle was injured in the right rectus abdominus to which *P. aeruginosa* was applied), transcriptional profiling identified 7 out of 136 differentially expressed genes that were involved in pyochelin biosynthesis, including the pyochelin receptor fptA, pchH, pchG, pchE, pchD, pchB, and pchC (the biosynthesis of Pch requires the iron-regulated *pchDCBA* operon) [106]. In addition, the iron-sulfur cluster genes were upregulated [111]. These findings suggest that iron uptake in this bacteria contributes to its pathogenic activity within infected wounds [111]. Other pathways that are involved in iron uptake in *P. aeruginosa* include the citrate-mediated Fe^3+^ uptake pathways that engages FecA, an outer membrane ferric citrate receptor, the FeoB transporter, and the PcoA, periplasmic ferroxidase [112]. The process of iron uptake from heme involves the TonB system which involves HasA, an extracellular heme-binding protein, HasR, and PhuR encoded on the *phuSTUVW* operon (a gene cluster encoding an outer membrane receptor and specific ABC transporters for heme and hemoglobin uptake) [113].

In addition to wound infections, *P. aeruginosa* infections in lungs are frequent in patients afflicted with cystic fibrosis [114]. In an effort to determine whether iron chelation may hinder the development of such infections in cystic fibrosis, administration of aerosolized bovine lactoferrin (bLF) was performed in a mouse model of cystic fibrosis with *P. aeruginosa* infection. Neutrophil numbers, pro-inflammatory cytokines, and microbial numbers were reduced with bLF treatment [115]. Although lactoferrin is considered a natural anti-microbial agent present in secretions of the airways, which also has the ability to bind iron [115], it is unclear whether its iron binding potential is responsible for the observed outcomes. Nonetheless, bLF may have potential as a clinical agent to alleviate pathogenic infections and inflammation in cystic fibrosis patients.

Manuka honey, produced from the nectar of *Leptospermum scoparium* (manuka bush), was discovered to elicit anti-microbial activity against multiple pathogens [116]. Specifically, the honey could hinder the growth of several pathogenic microbes including *P. aeruginosa*, *Escherichia coli*, and *S. aureus* [116]. Although the ferrozine-based iron chelation assay was utilized to determine that the honey mediates iron chelating activity [116] and the honey simulated an environment of limiting iron availability [116], it remains unclear whether the anti-microbial effect of the Manuka honey is due to its iron chelation ability. Further investigation is needed to not only identify the potential iron chelating component in the honey but to also further investigate whether mimicking an environment with low iron content may potentially diminish the growth of *P. aeruginosa*, which could potentially be utilized as a strategy to overcome antibiotic resistance.

### 3.2. Porphyromonas Gingivalis, Prevotella Intermedia, and Fusobacterium Nucleatum: Bacteria Associated with Periodontitis

Bacterial features—The oral microbiome can be composed of up to 700 species; an imbalance of these species could lead to the development of periodontitis [117]. Specifically, Porphyromonas gingivalis and Fusobacterium nucleatum are critically important in the amalgation of late and early colonizers within the oral cavity [117]. Furthermore, *P. gingivalis* and Prevotella intermedia are mutualistic in terms of heme acquisition, as described in detail below. *P. gingivalis* (Strain W83) is a Gram-negative oral bacteria with a genome size of 2,343,479 bp. P. intermedia is described as a anaerobe that is Gram-negative with a genome size (OMA14 strain) which is represented by two circular chromosomes of 2,280,262 and 867,855 bp, respectively [118]. Genome sequencing of five subspecies of F. nucleatum, a Gram-negative anaerobic microbe, has identified a range of genome sizes, namely 1.84–2.7 Mbp [119,120]. Within the periodontal pocket, in response to an altered microbiome, an inflammatory fluid is generated, called gingival crevicular fluid [121]; this exudate contains iron containing proteins such as hemoglobin, lactoferrin, and transferrin, which may contribute to the outgrowth of pathogenic oral bacteria [122].

Contribution of iron—Since *P. gingivalis* is deficient in siderophores [123,124] as well as specific heme precursor enzymes [125,126,127], it acquires heme from exogenous sources [128]. This bacteria acquires iron using (1) specific outer membrane receptors, (2) proteases, and (3) lipoproteins [128]. With respect to outer membrane receptors, *P. gingivalis* contains proteins that physically interact with hemoglobin (e.g., haptoglobin) and heme (e.g., hemopexin) in gingival crevicular fluid [129,130,131]. For proteases, specific genes identified in this bacteria include *rgpA* (which codes for gingipain that cleaves arginyl peptide bonds), *hagA* (which codes for hemagglutinin A), and *kgp* (which codes for gingipain that cleaves lysyl peptide bonds) [130,132]. One mutualistic behavior between *P. gingivalis* and *P. intermedia* involves the process of heme acquisition; specifically, this involves the HmuY protein in *P. gingivalis* and the proteolytic activity of *P. intermedia* [133]. The hemolytic activity of *P. intermedia*, which increases free hemoglobin, is thus proposed to provide an optimal growth environment for *P. gingivalis* [133,134]. In addition, mutualism via InpA (proteolytically oxidizing hemoglobin in *P. intermedia*), supports iron (III) protoporphyrin IX generation via hmuY from *P. gingivalis* [133,135].

Subgingival plaque *P. gingivalis* could be effectively inhibited in its growth rate and adhesiveness by the iron chelator, DFO, by reducing its ability to accumulate hemin, Fe^3+^-protoporphyrin IX, a virulence factor [136]. The effect of iron chelating agents were also investigated on *P. intermedia*, another Gram-negative microbe present in periodontal lesions [137]. DFRA could effectively inhibit its growth and biofilm-forming activities [137]. A blueberry extract, containing high levels of flavonoids which has potential to elicit iron chelating activity, was able to antagonize the growth, biofilm formation, and proteolytic activity (decreased matrix metalloproteinase secretion) of *F. nucleatum* [138]. The microbial activity of this microbe could also be opposed by bioactive components present in green and black tea (e.g., EGCG and theaflavins) which also appear to be associated with iron chelating activities [139].

Over the past two decades, there has been a rise in antibiotic-resistant infections, which could be attributed to the overuse of antibiotics (with evidence of country dependency [140]) and antibiotic resistance gene transfer to the bacteria present within the oral cavity [141,142]. Although the above described natural agents appear to elicit anti-microbial activities which may offer some protective health benefits against periodontitis, it remains unclear whether their effects are due to their iron-chelation ability. Thus, further investigation is needed to identify the iron chelating components in the blueberry extract and the tea as well as address their potential in mediating anti-microbial activities in these pathogenic microbes.

### 3.3. Streptococcus Pneumoniae: A Gram-Positive Bacteria

Bacterial Features—Infections that are due to *Streptococcus pneumoniae*, a pathogen with a core genome size of 1,536,569 bp [143], that causes pneumonia, meningitis, and bacteremia, is associated with multiple serotypes and thus a search for candidates to target as a treatment approach remains an ongoing effort [144].

Contribution of Iron—*S. pneumoniae* does not express siderophores, which is unlike other microbes [144,145]. To overcome this limitation, pneumolysin is released from *S. pneumoniae* (as a result of autolysin (a cell wall degrading enzyme activity [146])) to elicit hemolytic activity; this activity is responsible for the lysis of erythrocytes leading to the release of heme [147]. *S. pneumoniae* can acquire iron from hemoglobin and heme-binding proteins; specifically, receptors present on the surface of *S. pneumoniae* including pit1, pit2, and ABC transporters, are involved in the uptake of released iron from heme [148]. A mutant strain of *S. pneumoniae* that was defective in hemin uptake was found to diminish virulence in an intraperitoneally injected mouse model [145]. *S. pneumoniae* contains two operons, namely *piuBCDA* and *piaABCD* (prior naming system, *pit1BCDA* and *pit2ABCD*), which code for proteins involved in iron uptake [149]. *Pit1* and *pit2*, which are *S. Pneumoniae* loci which code for an ABC transporter, enables the Gram-positive bacteria to acquire iron from hemoglobin [148]. Using two different mouse models (pneumonia model in which *S. pneumoniae* was inoculated intranasally and a systemic model in which *S. pneumoniae* was inoculated via the intraperitoneal cavity), double knockouts of *pit* and *pit2* led to a marked reduction in *S. pneumoniae* virulence [148]. A proteomic study, using parallel metabolic pulse labeling in *S. pneumoniae*, was performed in the presence of the iron chelator, 2,2′-bipyridine, to limit iron content within the environment of the pathogen [144]. Under this condition, transport and binding proteins involved in *S. pneumoniae* pathogenesis as well as those involved in cell division (FtsA, FtsZ, and StkP) were downregulated [144]; in contrast, molecules involved in iron uptake were increased including PiuA (the lipoprotein component of ABC transporters) [81].

Altogether, these studies suggest that targeting the iron uptake pathways and/or use of iron chelators could antagonize the growth and virulence of *S. pneumoniae*.

### 3.4. Mycobacterium Tuberculosis

Bacterial Features—*Mycobacterium tuberculosis* is a pathogen with a genome size (H37Rv strain) of 44.1 Mbp [150]. This bacterium is predominantly intracellular and is the causative factor in tuberculosis, a highly contagious disease [151]. However, *M. tuberculosis* can also be disseminated extracellularly into the blood to secondary locations (e.g., central nervous and lymphatic systems) [151]. Current treatment regimens include a cocktail containing isoniazid, rifampicin, pyrazinamide, ethambutol, and streptomycin; regrettably, these agents can lead to adverse effects including liver damage and the development of *M. tuberculosis* resistant strains [151].

Contribution of Iron—Infections mediated by *M. tuberculosis* activate a host defense pathway that limits serum iron availability causing “anemia of chronic disease” [152]. To overcome this host limitation, *M. tuberculosis* has evolved mechanisms to acquire intracellular iron within host macrophages and myeloid dendritic cells via a siderophore-mediated process involving mycobactin and carboxymycobactin [152,153]. An endosome/lysosome metal ion transporter, NRAMP1 or natural resistance-associated macrophage protein 1, contributes to iron uptake in the bacterium that is located in phagosomes [152]. Thus, it has been suggested that this pathway could be a potential target for drug treatment against tuberculosis, a disease associated with increased resistance to current treatment strategies. A novel pyrazolopyridinone, PZP, which elicits intracellular iron chelator activity, could hinder the growth of *M. tuberculosis* [153]. Using in vivo guinea pig and mouse models with *M. tuberculosis* strains harboring loss of iron acquisition and uptake systems (located within the ESX-3 type VII secretion system) to restrict iron accessibility, the bacterial burden was markedly reduced [153].

Further work is needed to determine clinical efficacy of targeting the above described pathway in patients infected with *M. tuberculosis*.

## 4. Fungal Infections

Fungal Cell Structure—Since the fungal cell wall is the primary barrier that is encountered in response to anti-fungals, its characteristics have been investigated in an effort to understand its role in mediating anti-fungal resistance [154]. It is important to note that the fungal cell wall is comprised of two membrane components. The inner membrane is composed of glucans and chitin and provides not only structure but is tolerant of the immense internal forces arising from the fungal cytoplasm [154]. The outer membrane is composed of glycoproteins with both N and O-linked carbohydrates [154] and its composition may be altered under variable environmental conditions altering the ability of nutrients (including iron) to pass through to the plasma membrane [40].

Fungal Iron Uptake Pathways—The virulence and growth potential of fungi depend on specific metal ions, such as copper, zinc, manganese, nickel, and of relevance to this review, iron [155]. Although the iron metabolic pathway has been well delineated in *Saccharomyces cerevisiae*, a model organism [156], greater attention has been placed in understanding the iron pathways in pathogenic fungi including *Cryptococcus neoformans* and *Aspergillus fumigatus* with the past decade [40]. The mechanisms of iron uptake vary in different pathogenic fungi under divergent environmental iron conditions [157].

The most commonly described mechanisms across fungal species include the (1) reductive iron assimilation (RIA) and (2) non-reductive (siderophore-mediated) iron uptake [158]. The RIA process involves reduction of ferric iron to the ferrous form via ferrireductases (e.g., *FRE* genes); this is then followed by its uptake into the cells by the permease FTR1 along with the oxidation of the iron via the activity of a ferroxidase (Fet3) [159]. With respect to *S. cerevisiae*, it primarily employs both low-affinity and high-affinity non-reductive iron transport systems in conjunction with metalloreductases (which reduce iron) and siderophore mediated iron transport mechanisms [40,41,157]. Many fungal species utilize siderophores in iron acquisition; such fungi transport iron-bound siderophore complexes via transmembrane transporters and multivesicular bodies [40,159]. These siderophores are concentrated within the fungal cell wall and within the fungal periplasmic space [40]; the presence of cell wall glycoproteins (e.g., FIT) enables the iron uptake via a siderophore-mediated process [40]. The majority of fungal siderophores are classified into two categories: (1) hydroxamates (e.g., rhodotorulic acid, coprogens, ferrichromes, and fusarinines) and (2) polycarboxylates [157]. Additional sources of fungal iron include hemin [157], heme, and hemoglobin which involve an iron uptake pathway that differs to the ones described above [159]. Specifically, a family of proteins which contain a cysteine-rich Common in Fungal Extracellular Membrane (CFEM) domain are involved (e.g., Rbt, Pga7, and Csa2) in *C. albicans* and *S. cerevisiae* and components of the endosomal sorting (ESCRT-I) complex are involved in *C. neoformans* [159].

Due to the vast array of iron acquisition mechanisms employed by fungi, these pathways involved in iron accumulation contributing to fungal virulence could be potentially targeted to formulate novel treatment strategies.

### 4.1. Cryptococcus Neoformans

Fungal Features—*Cryptococcus neoformans*, a basidiomycete fungus involved in meningitis, can lead to a poor outcome in patients who are immunocompromised [160]. The treatments are limited to anti-fungal agents such as amphotericin B (which binds to sterol in fungal cell membranes, leading to pore formation and ultimately fungal death [161]) and fluconazole (which disrupts the fungal membrane and ergosterol synthesis) [162]). However, these drugs elicit side effects including kidney damage [160] and thus, other agents are needed to improve treatment responses.

Contribution of Iron—It is well established that the iron permease (Cft1) and ferroxidase (Cfo1) are involved in the iron uptake pathway in *C. neoformans* [160]. Cft1 mutant strains of this fungi leads to reduced growth, reduced intracellular iron, and elevated susceptibility to miconazole and amphotericin B [163].

In a mouse model, intranasal inoculation of *C. neoformans* containing a mutation in *cft1* markedly reduced fungal virulence [163]. Intranasal instillation of *C. neoformans* with a deficiency of *Cfo1* (but not *Cfo2*) into mice led to a reduction in virulence but also increased the sensitivity to amphotericin B and fluconazole [164]. The mechanism underlying this finding was suggested to be due to reduction of the cofactor, heme, which is needed for enzymes involved in ergosterol biosynthesis such as Erg11, an anti-fungal drug target [164]. Further, the addition of heme or ferrioxamine (a siderophore) reduced the drug sensitivity (amphotericin B and fluconazole) in the microbe [164]. In support, decreased virulence of *C. neoformans* was noted with genetic deficits in these fungal iron uptake proteins coinciding with reduced resistance to the anti-fungal agents [160,163,164].

Chelation of intracellular iron with bathophenanthroline disulfonate (BPS) or DFP, in combination with fluconazole or miconazole, synergistically altered the growth capacity of *C. neoformans* in vitro [160]. Based on these findings, the use of iron chelators or targeting the above described iron mediators may offer alternative strategies of treatment as adjuvant drugs along with the anti-fungals [160].

### 4.2. Aspergillus Fumigatus

Fungal Features—*Aspergillus fumigatus*, an airborne saprophytic fungus that is responsible for the development of invasive pulmonary aspergillosis, is common in patients characterized by iron overload and with blood cancers [165]. First-line treatment regimens include the application of systemic antifungals such as voriconazole and isavuconazole; however, resistance to these agents has been reported [166].

Contribution of Iron—Since *A. fumigatus* is unable to acquire host iron directly, specifically from transferrin, ferritin, or heme, it has developed efficient iron uptake mechanisms including (1) RIA and (2) siderophore-mediated processes [167]. As described earlier, RIA involves reducing Fe^3+^ to Fe^2+^ via ferrireductases that are present within the fungal cell membrane; the imported iron is then oxidized by a ferroxidase and a permease, FetC and FtrA, respectively [168]. *A. fumigatus* is also capable of acquiring iron via extracellular siderophores, fusarinine C (FsC) and triacetylfusarinine C (TAFC), which are then imported into the fungus via transporters in the membrane (SIT, siderophore iron transporter) [169]. Although genetic inactivation of RIA does not alter fungal virulence, SidA, which is a critical enzyme in the biosynthesis of siderophores, was necessary for mediating virulence in a murine model involving intranasal instillation [169]. The biosynthesis of siderophores in *A. fumigatus* is regulated by the GATA transcriptional regulator, SreA [170]; however, *A. fumigatus* deficient in SreA does not differ to the wild type strain in terms of virulence [170]. Using two pulmonary invasive aspergillosis murine models (leucopenic mice that are immunosuppressed with cortisone acetate and cyclophosphamide as well as a non-leucopenic model that is immunosuppressed with cortisone acetate), the deficiency of HapX in *A. fumigatus* led to a reduction in the spread of the fungal infection [171]. HapX is a bZip (basic leucine zipper containing domain) transcriptional regulator involved in downregulating iron-dependent metabolic pathways and the biosynthesis of heme [171].

The combination of the iron chelator DFRA, with a liposomal preparation of Amphotericin B, was effective in reducing fungal infections in murine models of pulmonary Aspergillosis which supported murine survival [165]. Although this study, which utilizes an iron chelating treatment strategy for this disease, shows promise in an in vivo mouse model, further work is needed to determine clinical feasibility of using such iron chelators in patients infected with *A. fumigatus*.

### 4.3. Rhizopus Oryzae

Fungal Features—*Rhizopus oryzae* is a filamentous fungus involved in the development of mucormycosis, a common infection in (a) patients with diabetic ketoacidosis [172], (b) those who are immunocompromised as a result of cytotoxic chemotherapy [173], or (c) those undergoing organ transplantation [174,175]. Current treatments include Amphotericin B, as described above, which often leads to kidney damage [176]. Unfortunately, due to surgical disfigurement and the high mortality index, the development of improved treatment regimens remain essential [177].

Contribution of Iron—The genome sequencing project for *R. oryzae* has identified several genes involved in iron uptake including three ferric reductases, six copper oxidases, a high-affinity iron permease, siderophore permeases, SreA, and genes involved in the uptake of iron from heme [178,179]. The fungal receptors, FOB1 and FOB2 (ferrioxamine binding plasma membrane proteins) are involved in promoting the binding of ferrioxamine, a siderophore, [180] in order to promote iron uptake via the FTR1 permease mechanism [181]. Loss of FTR1 via genetic manipulation (via RNAi and reduction in DNA copy number) decreases iron acquisition in *R. oryzae* as well as fungal infections in a murine model of diabetic ketoacidosis infected with spores via the tail vein or intranasal instillation [181].

In a diabetic ketoacidosis mouse model and a DFO-treated mouse model injected with *R. oryzae* spores into the tail vein, *FOB1* and *FOB2* deficient fungi were essential for mediating virulence in the DFO-model only [180]. Furthermore, these genes were critical for mediating iron uptake into *R. oryzae* [180]. However, other iron chelators, such as DFP and DFRA, were successful in reducing virulence and improved survival in an in vivo diabetic ketoacidotic mouse model [182,183]. It is suggested that the acidotic condition of the diabetics may contribute to decreased binding capacity of iron to transferrin [177], increasing free iron levels to promote mucormycosis infections [172]. Treatment with DFO increased the infectivity of *R. oryzae* in immunocompetent guinea pigs [184] and albino guinea pigs [185].

The oral administration of DFRA, an iron chelator, resulted in clinical improvement in a 40-year old patient infected with Rhinocerebral mucormycosis (an opportunistic invasive fungal infection) in combination with liposomal amphotericin B [186]. This one clinical study warrants further clinical application of DFRA or other iron chelators in the treatment of patients infected with *R. oryzae*. Collectively, targeting these fungal iron pathway holds promise to improving existing therapeutic modalities for overcoming the detrimental health consequences of *R. oryzae* fungal infections.

## 5. Concluding Perspectives

Although limited applications for iron chelation therapeutic approaches are noted in clinical practice for the topics presented herein, evidence from in vitro and in vivo animal models, which cover a wide range of diseases, provides a strong positive foundation for future clinical applications. With respect to iron availability, it is essential that iron levels in the host be tightly controlled to hinder the development of microorganismal infections within the host by (1) supporting the capacity of iron-binding proteins in the host plasma to limit iron availability to hinder the growth and virulence of microorganisms and (2) diminishing the intracellular host iron by targeting TBI or NTBI uptake processes, increasing ferritin-bound iron/reducing ferritinophagic processes, and increasing FPN-mediated iron export. Although there is evidence for iron chelators in hindering microorganismal virulence, this is an area that would benefit from further research investigation. Furthermore, the identification of the iron chelating components in the natural compounds derived from plants (e.g., blueberry extract, tea, as well as honey) would also be beneficial in the identification of naturally occurring iron chelators.

## Abbreviations

ABC	ATP-binding cassette
AIDS	acquired immune deficiency syndrome
ALT	alanine aminotransferase
ART	anti-retroviral therapy
ATPase ABCE1	ATP binding cassette subfamily E member 1
bLF	bovine lactoferrin
NRAMP1	natural resistance-associated macrophage protein 1
BLM	bleomycin
BPS	bathophenanthroline disulfonate
bZIP	basic leucine zipper containing domain
CD81	Cluster of Differentiation 81
CDK	cyclin dependent kinases
CFEM	Common in Fungal Extracellular Membrane
CLDN1	Claudin 1
DCYTB	duodenal cytochrome b
DFO	Desferioxamine or deferoxamine
DFP or L1	Deferiprone
DFRA or DFX	Deferasirox
DFT	Desferrithiocin
DMT1	Divalent Metal Transporter 1
DNA	deoxyribonucleic acid
EGCG	Epigallocatechin-3-gallate
EGFR	epidermal growth factor receptor
eIF3	Eukaryotic initiation factor 3
FDA	Food and Drug Administration
Fe-SIH	iron-salicylaldehyde isonicotinoyl hydrazine
FeSO_4_	iron (II) sulfate
FPN1	ferroportin
FsC	fusarinine C
FTN	ferritin
Fur	ferric uptake regulator
GATA	Globin Transcription Factor
HAMP	hepcidin
HasA	heme acquisition system A
HCP1	Heme Carrier Protein
HCV	Hepatitis C Virus
HIV	Human Immunodeficiency Virus
HO-1	heme oxygenase 1
HO-2	heme oxygenase 2
IFN	Interferon
IRES	internal ribosome entry site
JAK	Janus Kinase
LIP	labile iron pool
NF-kB	nuclear factor kappa-light-chain-enhancer of activated B cells
NS3	non-structural protein 3
NTBI	non-transferring bound iron
OCLN	Occludin
OMP	outer membrane protein
PBL	peripheral blood lymphocytes
PCFT	proton-coupled folate transporter
Pch	pyochelin
Pvd	pyoverdine
PZP	pyrazolopyridinone
RBC	red blood cells
RIA	reductive iron assimilation
RNA	ribonucleic acid
RNR	ribonucleotide reductase
ROS	reactive oxygen species
SIT	siderophore iron transporter
SLC39A8	Solute carrier family 39 member 8
SRB1	Scavenger receptor class B type 1
STAT	Signal Transducer and Activator of Transcription
STEAP3	Six Transmembrane Epithelial Antigen of Prostate 3
TAFC	triacetylfusarinine C
TBI	transferrin-bound iron
TGF-β	transforming growth factor beta
